# Determinants of change in accelerometer‐assessed sedentary behaviour in children 0 to 6 years of age: A systematic review

**DOI:** 10.1111/obr.12882

**Published:** 2019-06-27

**Authors:** Liane B. Azevedo, Esther M.F. van Sluijs, Helen J. Moore, Kathryn Hesketh

**Affiliations:** ^1^ School of Health and Social Care Teesside University Middlesbrough UK; ^2^ MRC Epidemiology Unit and Centre for Diet and Activity Research University of Cambridge Cambridge UK; ^3^ UCL Great Ormond Street Institute of Child Health London UK; ^4^ Wolfson Research Institute for Health and Wellbeing Durham University Durham UK

**Keywords:** behavioural change technique, early years, objectively‐measured, socioecological model

## Abstract

Sedentary behaviour tracks from early to middle childhood, suggesting the need to intervene early. The aim of this systematic review was to identify determinants of change in accelerometer‐assessed sedentary behaviour in young children, with a view to informing interventions. Ten electronic databases were searched. Longitudinal and intervention studies were included if they (a) targeted sedentary behaviour in young children (less than of equal to 6 years), (b) assessed change in accelerometer‐assessed sedentary behaviour, and (c) reported on at least one determinant of change in sedentary behaviour. Intervention components were coded according to clusters of behaviour change technique (BCT) (ie, grouping similar BCTs components). Data synthesis was guided by the socioecological model. Sixteen studies (four longitudinal; 12 intervention) met the inclusion criteria. Two (out of five identified determinants) were associated with an increase in sedentary behaviour in longitudinal studies: the after childcare/school period and transition from childcare to school. Three (out of 21 identified determinants) were associated with a decrease in sedentary behaviour in intervention studies: “goals and planning” (ie, “behavioural contract”), “repetition and substitution” (ie, “graded tasks”), and “reward and treat” (ie, “incentives”). The environmental and interpersonal determinants identified in this review may help to inform behavioural strategies, timing, and settings for future interventions.

## 1 INTRODUCTION

There is growing evidence that the most efficient and cost‐effective way to prevent health problems is to intervene in early life before behaviour and health patterns have been firmly established.^1^ Although there is a general perception that young children are spontaneously active, a review examining levels of accelerometer‐assessed sedentary behaviour in children 0 to 6 years old revealed high levels of sedentary time in this age group.^2^ They found that children spent a median of 77% of the day (range across studies 34% to 94%) or approximately 10 hours sedentary.

Sedentary behaviour, defined as any waking behaviour characterized by an energy expenditure less than or equal to 1.5 metabolic equivalents (METs), while in a sitting, reclining, or lying posture,^3^ has been associated with obesity in children and young people.^4^, ^5^, ^6^ It is unclear whether this association is causal,^7^ and interventions targeting sedentary behaviour in children and young people (0 to 17 years old) have only shown small and clinically irrelevant effects on BMI reduction.^8^ This is however a complex field as sedentary behaviour is frequently targeted alongside with other behaviours (eg, diet and physical activity) in a multibehaviour approach to prevent and treat obesity.^7^

In addition to overweight and obesity, sedentary behaviour in school‐age children has been associated with a range of other negative health effects including a higher clustered cardiometabolic risk score, lower fitness, unfavourable behavioural conduct, and lower self‐esteem.^9^ Although there is little evidence about the role of sedentary behaviour on developmental outcomes in the early years, certain screen‐based sedentary behaviours may have no benefit and potential to harm motor and cognitive development.^10^ It is however important to recognize that sedentary behaviours such as reading has well‐known benefits for cognitive development^11^ and parent‐child interaction.^12^

Sedentary behaviour appears to track at moderate to high levels from early to middle childhood years.^13^, ^14^ This suggests that benefits of early intervention to reduce sedentary behaviour may be carried over into school age, where evidence of the health benefit reducing sedentary behaviour for health is more robust.^9^ Establishing the determinants of a behaviour in early life is therefore important in order to intervene effectively.^15^ Determinants of sedentary behaviour have been investigated previously in a systematic review of children up to 18 years old.^16^ However, evidence was limited for our population of interest (less than or equal to 6 years), including only one study with very young children (toddler and preschool age).^17^ This study provided a proxy report of sedentary behaviour (parent self‐reported) and accelerometer‐based data. In this age group, proxy‐reported questionnaires are commonly used to assess sedentary behaviour due to cognitive limitations of young children. However, the use of self‐report for sedentary behaviour, usually restricted to screen time, has been criticized as it accounts for only a small proportion of the sedentary behaviour that children engage in.^18^, ^19^ Parents' proxy‐reported sedentary behaviour might also be influenced by social desirability and recall bias especially due to the intermittent and incidental nature of children's sedentary behaviour.^20^ This is particularly true in young children where sedentary behaviour includes being restrained in a car seat, high chair, or pushchair.

This systematic review is part of a collection of reviews that aim to explore the determinants of obesity‐related behaviours in young children (eg, diet, physical activity, and sedentary behaviour).^21^, ^22^, ^23^ The aim of this review is to identify determinants of change in accelerometer‐assessed sedentary time in young children (0‐6 years old), with a view to informing interventions. Determinants will be organized according to the social ecological model as done previously.^21^, ^23^ The socioecological model provides a useful framework for identifying potential determinants at individual (ie, age, weight status, and sedentary behaviour at baseline), interpersonal (ie, family, carers, and teachers) environmental (ie, playground density and equipment), and policy (ie, safe places to cross roads and longer lunch breaks) levels. The socioecological model recognizes that individuals are embedded within a large interactive social system, which has a cumulative effect on health outcomes.^24^, ^25^ The use of this framework will allow us to identify the level‐specific determinants of sedentary behaviour. Additionally, it will use the taxonomy of behaviour change techniques to code the content of behavioural interventions.^26^ The use of BCT taxonomy is included with a view to gathering knowledge to guide future research and implementation by reporting the “active ingredients” of interventions with precision.

## 2 METHODS

This systematic review is reported according to the Preferred Reporting Items for Systematic Reviews and Meta‐Analyses (PRISMA) criteria.^27^ The protocol for the overall systematic review process has been registered in the International Prospective Register for Systematic Reviews (PROSPERO), registration number CRD42012002881.

As stated earlier, this systematic review is part of a suite of reviews to explore the determinants of obesity‐related behaviours.^21^, ^22^, ^23^ A detailed protocol including study design, search, and quality assessments strategies has been published elsewhere.^28^ This review deviates from the overall protocol with respect to the following inclusion criteria: (a) exclusion of cross‐sectional studies; (b) exclusion of subjective measures of sedentary behaviour; and (c) exclusion of diet and physical activity search terms (an example of the search strategy^28^ is presented in Data S1). One deviation from protocol on this present review was also present in another systematic review from this collection (ie, exclusion of cross‐sectional studies).^21^ Other changes were particular in this review including exclusion of subjective measures and narrowing of search terms. Cross‐sectional studies were excluded as it can be difficult to make casual inference, which is the aim of this review. Therefore, to establish the longitudinal predictors (ie, determinants) of change in sedentary behaviour and to provide evidence on how to effect positive behaviour change, only longitudinal and intervention studies were included.

Studies with subjective measured sedentary behaviour were searched and sifted up to the full‐text stage. However, there was a high heterogeneity of methods used (eg, self‐ and proxy‐reported questionnaires and diaries). Moreover, self‐reported measurements tend to be restricted to TV viewing, which is a small proportion of young children's sedentary behaviour: children can also spend long periods engaged in nonscreen sedentary behaviours (eg, restrained in a car seat, high chair or pushchair, colouring, and doing puzzles). Therefore, studies only reporting on subjective measures of sedentary behaviour as an outcome were excluded and accelerometer‐assessed sedentary behaviour were included as a more accurate measurement of the behaviour.

### 2.1 Search strategy

A systematic search was undertaken in March 2018 in 10 electronic databases: MEDLINE; EMBASE; CINAHL; PsycINFO, Applied Social Sciences Index and Abstracts (ASSIA); Sociological Abstracts (via Proquest); British Nursing Index (BNI); Web of Knowledge; Education Resources Information Center (ERIC); and Sports Discus. No date or language restrictions were applied. Files were imported into EndNote reference management software (version X7.01, Thomson Reuters), and duplicates were removed. References of included articles and relevant reviews identified in the search were hand searched for additional relevant publications.

### 2.2 Study selection

For quality control, two batches of titles and abstracts (570 in total) were screened for inclusion by four reviewers. Disagreements were discussed until consensus was achieved. Since discrepancies between reviewers were low (less than 5%), the lead reviewer (L.A.) screened all remaining titles and abstracts. Full texts were subsequently obtained and read in full; eligibility for inclusion was assessed independently by two reviewers. Discrepancies were resolved by discussion or by consultation with a third reviewer until consensus was reached.

### 2.3 Study inclusion and exclusion criteria

Studies were included if (a) children aged 0 to 6 years old (at baseline) were included as the population of the study; (b) assessed a within‐child change in accelerometer‐assessed sedentary behaviour as an outcome; (c) had a longitudinal or intervention design (either randomized and nonrandomized trials); (c) assessed at least one identifiable determinant of sedentary behaviour at individual, interpersonal, environmental, or policy level; and (d) for intervention studies, explicitly targeted sedentary behaviour or sedentary activities (such as screen‐based activities or sitting), following the definition of sedentary behaviour (ie, waking behaviour characterized by an energy expenditure less than or equal to 1.5 METs, while in a sitting, reclining, or lying posture).^3^

Studies were excluded if they (a) involved clinical populations (eg, children with cerebral palsy, cystic fibrosis, and autism); (b) were performed in laboratory settings; (c) targeted active video gaming; (d) studies that referred to “failure to meet a physical activity guideline” as a definition for sedentary behaviour, and (e) for intervention studies, had no control group.

### 2.4 Quality assessment

Study quality was evaluated using assessment tools specific to each study design, published by the Evidence for Policy and Practice Information (EPPI) centre.^29^ The quality assessment criteria are specified in Table 1. Studies were classified according to the number of criteria met (intervention: maximum 8; longitudinal: maximum 6). Quality was judged as follows: for intervention studies: low: less than or equal to 2; intermediate: 3 to 5; or high: greater than or equal to 6; and for longitudinal studies: low: less than or equal to 2; intermediate: 3 to 4; or high: greater than or equal to 5. Quality assessment was performed independently by two reviewers and any disagreements resolved by a third reviewer.

**Table 1:** Evidence for Policy and Practice Information (EPPI) quality assessment criteria by study design

Type of Study	Assessment Criteria
Intervention studies	Randomization
	Effect of intervention reported for all outcomes
	Preintervention data on all outcomes
	Postintervention data on all outcomes
	Allocation concealment
	Blinding
	Objective measurement of outcome
	Retention greater than 70%.
Longitudinal studies	More than 50 participants analysed
	Study represent general population
	Prospective study design (versus cross sectional)
	Multivariate analyses (versus univariate)
	Objective (versus subjective) measure of outcome
	Objective measure of exposure.

Note: Each criterion was scored as yes (1) or no (0).

### 2.5 Data extraction

A standardized data extraction form was piloted, completed by one reviewer, and checked by a second reviewer. The following information was extracted by reviewers: study information (eg, author and year); baseline descriptive characteristics; study design; setting; sedentary behaviour measurement and outcomes; methods of analysis; follow‐up (duration, sample, and results); and potential determinants and their association with the outcome. For all studies, the latest follow‐up data available before the children were 6 years old (or as close to as possible afterwards) were included. If results were stratified by specific times of the day, data for the largest time periods were extracted. For intervention studies, all factors targeted in the intervention (eg, parental knowledge and parental modelling) were extracted as potential determinants of change in sedentary behaviour. To score these determinants, the difference in change in sedentary behaviour between control and intervention groups over time was assessed. This was deemed to provide evidence of factors targeted in interventions (ie, determinants), which were associated with change in the outcome. Where possible, results of multivariable rather than univariable models were included.

### 2.6 Data synthesis

Because of heterogeneity across studies (including design, setting, measures of determinants, and analysis type), a meta‐analysis was not appropriate. A narrative synthesis and harvest plot analysis were therefore undertaken.

Determinants of sedentary behaviour from intervention and longitudinal studies were broadly classified across four levels of the socioecological model^24^: (a) Individual (child); (b) Interpersonal (parent/caregiver); (c) Environment (home, school, and childcare); and (d) Policy (government). Concerning childcare (environment level of the socioecological model), in this paper, the term is used to describe the period before starting formal school. For the intervention studies only, the Behaviour Change Technique Taxonomy (v1), comprising 93 Hierarchically Clustered Techniques,^26^ was also used to identify and cluster BCT applied. Information on protocols of included papers were also examined. The BCT coding was performed by one reviewer and verified by two others; in case of discrepancies, they were resolved through discussion.

Consistency regarding the association of each determinant from longitudinal and intervention studies with accelerometer‐assessed sedentary behaviour was summarized according to Sallis et al.^30^ The consistency of association was based on the percentage of reported findings that supported the hypothesized association as follows: “0” (no association) if supported by 0% to 33% of individual studies, “?” (inconsistent evidence) if supported by 34% to 59%, and “+” or “−” if supported by 60% to 100%. Where four or more studies reported on a potential determinant, double signs were used to indicate greater confidence (eg, “00,” “??,” “++,” and “−−”). For intervention studies, consistency was analysed at BCT component level and cluster level.^26^ According to the Behaviour Change Technique Taxonomy (v1), the BCT components were organized hierarchically into 16 clusters, which were conceptually coherent BCTs including (a) social support, (b) regulation, (c) feedback and monitoring, (d) associations, (e) repetition and substitution, (f) antecedents, (g) shaping knowledge, (h) self‐belief, (i) scheduled consequences, (j) reward and threat, (k) goals and planning; (l) comparison of outcomes; (m) identity, (n) natural consequences, (o) comparison of behaviour, and (p) covert learning.

Finally, each study was presented as a bar chart and summarized using the harvest plot format.^31^ The harvest plot emulates the visual representation of a forest plot providing evidence between the competing hypothesis (no change and positive or negative change), weighted by study quality and sample size.

## 3 RESULTS

**Figure 1:**
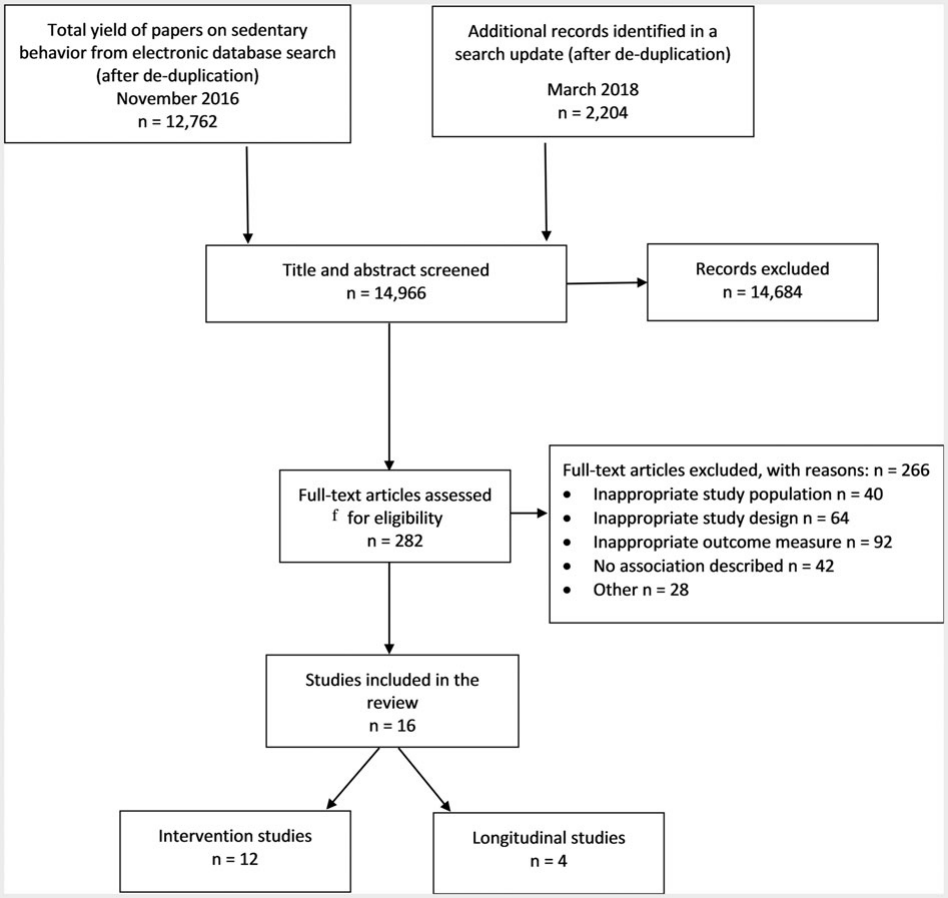
Selection of studies for inclusion in the systematic review

A total of 14 966 references were retrieved, of which 282 were read in full, and 16 studies (four longitudinal and 12 intervention studies) met the inclusion criteria (Figure 1). Sixty‐one studies were excluded because of proxy or self‐assessed sedentary behaviour.

### 3.1 Summary of study characteristics

Across the 16 included studies, a total of 12 495 individuals were included. Eight studies were conducted in Europe^32^, ^33^, ^34^, ^35^, ^36^, ^37^, ^38^, ^39^ (of which three were in the United Kingdom^36^, ^37^, ^38^), five in North America,^40^, ^41^, ^42^, ^43^, ^44^ and three in Australia.^45^, ^46^, ^47^ Nine of the 16 studies were published in or after 2015.^33^, ^34^, ^35^, ^39^, ^40^, ^42^, ^44^, ^46^, ^47^ Only one study included children younger than 3 years old.^47^

Ten studies used various Actigraph models to assess sedentary time,^32^, ^33^, ^34^, ^35^, ^36^, ^37^, ^38^, ^39^, ^40^, ^42^, ^45^, ^46^ three studies used Actical,^43^, ^44^, ^45^ and Activpal and Actiheart were used in one study each.^41^, ^47^ Different cut‐points were used to define sedentary behaviour,^48^, ^49^, ^50^, ^51^, ^52^, ^53^, ^54^, ^55^, ^56^ varying from 100 cpm^50^, ^52^, ^53^, ^54^, ^55^ to 1592 cpm.^49^ One study^40^ used activity energy expenditure (AEE) and physical activity ratio (PAR) as a cut‐off between sedentary behaviour and light activity.^48^ Only four out of the nine cut‐points applied were established and validated in a preschool population.^48^, ^49^, ^51^, ^52^

#### 3.1.1 Longitudinal studies

The main characteristics and findings of the longitudinal studies are summarized in Table 2. A total 1454 children took part in the four included studies. Follow‐up duration varied between 1 and 3 years. Overall, sedentary time increased significantly over time in two studies^45^, ^46^ and remained stable in the other two studies.^34^, ^41^

**Table 2:** Summary of the included longitudinal studies

Author, Year, and Country	Population/Setting	Duration	Outcome (Accelerometer, Valid Days, Cut‐points)	Main Finding
Arundel et al, 2013, Australia^45^	Population: Age: 5 to 6 y Girls n = 295 (48%), boys n = 313 (52%) Maternal education used as proxy measure of SES (level of education): low 33%, medium 35%, and 32% high. Setting: Data from the CLAN and the Health, Eating, and Play Study (HEAPS)22	3 and 5 y	Actigraph 7164; 1 min epoch; cut‐point less than or equal to 100 cpm (Trost et al, 2002); valid days—3 weekdays after school and whole day (greater than or equal to 610 min [T1], greater than or equal 647 min [T2], greater than or equal 635 min [T3]; greater than or equal 20 consecutive minutes of zero counts)	Significantly increase afterschool sedentary time over 3 years Three years, boys: 8.37 (95% CI, 6.3‐10.41), *P* < .001; girls: 5.36 (95% CI, 3.37‐7.34), *P* < .001.
Carson et al, 2016, Australia, Happy Study^46^	Population: Age: 4.2 ± 0.7 y (3 to 5 y) N = 177 (56.5% male) 79.7% born in Australia 20.3% born in other countries. Participants recruited from areas of lowest socioeconomic quintile, medium, and high based on the SEIFA Setting: Day cares and preschools	1 y	Actigraph GT1M, nonwear time defined as as greater than or equal to 10 min of consecutive zeros. Cut‐point of less than 100 counts/min or less than 25 counts/15‐s defined as sedentary (Janssen et al, 2013). Participants were required to have 50% of wear time for the during childcare/school period.	Increase sedentary time in Transition from childcare to school (34‐54 min/d or 2%‐3% wear time)
Janz et al, 2005, United States, The Iowa Bone Development Study^41^	Population: Age: 5.6 ± 0.5 y 176 boys and 202 girls, 95% white Setting: Community	3 y	Actiheart, model 7164, 8 h per day greater than or equal to 3 d. Puyau et al (2002) inactive minutes were defined by a cut‐point of 1.4 METs	Sedentary behaviour stable during middle childhood. No significant change in inactivity mean rate between boys and girls Mean rate difference boys: 25.4 S, SD = 18.0; Girls = 26.0, SD = 18.2; mean rate difference (boys‐girls): −0.6 CI (mean rate difference): −4.2 to 3.1
Michels et al, 2016, Switzerland, Ballabeina Study^34^	Population: Age: 3.9 to 6.3 y N = 291 47% boys; 76.4% had one parent born outside of Switzerland Setting: Childcare in France, Germany, and Switzerland	1 y	Actigraph, 15 s epoch; 3 d—6 h, 10 min consecutive zeros; cut‐points: less than or equal to 25 counts	No significant association between sedentary time and total QOL (emotional, social, and school). Sedentary time: Total QOL: B = −0.058, *P* = .581; Emotional QOL: B = −0.002, *P* = .982; Social QOL: B = −0.012, *P* = .907; School QOL: B = −0.088, *P* = .403

Abbreviations: CLAN, Children Living in Active Neighborhoods; QOL, quality of life; SEIFA, Socio‐Economic Indexes for Areas; SES, social economic status.

#### 3.1.2 Intervention studies

The main characteristics and findings of the intervention studies are summarized in Table 3. A total of 11 041 children took part in the 12 included studies. Intervention duration was 6 months or longer for half of the studies^33^, ^35^, ^38^, ^39^, ^40^, ^43^ (N = 6); other studies had a shorter intervention duration. Eight of the 12 intervention studies evaluated interventions with an emphasis on both physical activity and sedentary behaviour.^32^, ^33^, ^36^, ^37^, ^38^, ^40^, ^44^, ^47^ Three studies included an additional diet focus^35^, ^39^, ^43^; only one study^42^ solely targeted sedentary behaviour. Parents were the targeted agents of change in all but two studies.^32^, ^36^ Two studies showed a significant intervention effect on accelerometer‐assessed sedentary behaviour^37^, ^44^; all others showed no effects.

**Table 3:** Summary of the included intervention studies

Author, Year, Country, Study	Population/Setting	Sedentary Behaviour Intervention/Other Behaviours Targeted	Duration	Outcome (Accelerometer, Valid Days, Cut‐points)	Main Findings
Adamo et al, 2017, Canada, ABC trial study^40^	Population: Age: 3 to 5 years Intervention: CC: 49 children (six childcare centres); age: 3.5 ± 0.4 y, 39% males; household income: 63% > $100 000; 23% $30 000 to $99 999; 15% < $30 000.CC + Home: 71 children (six childcare centres); age: 3.8 ± 0.6 y; 72% males; household income: 53% > $100 000; 24% $30 000 to $99 999; 16% < $30 000. Control: N = 56; age: 3.5 ± 0.5 y; 42% males, household income: 86% > $100 000; 8% $30 000 to $99 999; 6% < $30 000. Setting: Childcare	The study used the socioecological model of health promotion. This was a childcare‐specific intervention, which was based on an evidence‐based train‐the‐trainer approach. The ABC resource training manual outlined the activity program with log sheets to track daily activities, the MusiGo (http://themusigokids.com/). Researchers provided educators with weekly schedules suggesting a set of activities from the ABC manual that could be incorporated into the daily curriculum. The aim of the physical activity activities was to help children to meet physical activity and sedentary time guidelines. Other behaviours: PA	6 mo	Actical accelerometers (MiniMitter Co., Inc, Bend, Oregon); 15‐s epoch; weekdays with at least 4 h of accelerometer wear time during childcare centre hours (from 8:30 am to 4:30 pm) and at least 1 h of wear time during outside childcare hours. Cut‐points—Adolph et al (2012) for preschool‐aged children (less than 0.015 kcal kg^‐1^ min^‐1^ or PAR of 1.6.	No significant difference in sedentary behaviour between intervention groups. Mean difference: CC vs COM: 0.2 (95% CI, −1.3 to 1.7), *P* = .810; CC + Home vs COM: 00; (95% CI, 1.9‐1.9), *P* = .995
Cardon et al, 2009, Belgium^32^	Population: Age: 5.3 ± 0.4 yTotal sample: 52%boysIntervention: play equipment: 145; marking: 147; play equipment + marking: 145;Control: 146 Setting: Preschool	The study explored if providing play equipment (eg, skippy balls, soft throwing discs, tail balls, spider balls, throwing rings, funny‐shaped balls, sets of aiming rings, bean bags, hoops, soft grab balls, soft balls, coloured wipes, and jumping bags), and painting marking was effective to increase physical activity and reduce sedentary behaviour in the preschool playground. Preschool teachers made the equipment available during recesses. Research team did the marking. There were three conditions: (a) play equipment provided during break time; (b) markings painted on the playground; and (c) play equipment was provided, and markings were painted. 4 to 6 wk of intervention Other behaviours: PA	6 wk	GT1M Actigraph, 15‐s epoch, cut‐points (Sirad et al, 2005, [SED: less than 364 for 4‐year‐olds; less than 399 for 5‐year‐olds]), children used during recess time	No significant change in sedentary behaviour during recess time. Play equipment: β = 2.1(*SD* 2.6); marking: β = −1.1(*SD* 2.6); play equipment + marking: β = −1.7(*SD* 3.6)
De Craemer et al, 2016, Belgium, ToyBox study^33^	Population: Age: 4.4 ± 0.6 y Intervention: N = 529 (54.6% boys); age: 4.4 ± 0.6 yControl: 330 (53.90% boys); age: 4.3 ± 0.6 y Setting: Preschool	The ToyBox intervention had a structured sedentary behaviour module. The module focused on children reducing sitting time at school, home, or leisure time, and teachers were asked to use the material for at least 1 h per week and performed the activities that were listed in a classroom activity guide The module constituted of a story book told to children, a handbook of classroom activities to support short and long breaks, instructions for teachers with activities that could be done while standing up, and teachers training. Parents were provided with newsletters explaining about sedentary behaviour and how to reduce sedentary behaviour; tip cards with activities that could help children to change sedentary behaviour and posters with key messages to reduce sedentary behaviour. Other behaviours: PA	7 mo	Actigraph GT1M and GT3X, GT3X+, 15‐s epoch, valid days—nonwear 10 min consecutive zeros, 6 h per day, two weekdays and one weekend. Evenson et al (2008) cut points	No effect on objectively measured sedentary time. Total SB B = −0.93 (95% CI, −2.30 to 0.43), *P* = .18. However, subgroup analysis (gender, SES, and SED at baseline) was significantly different
Hinkley et al, 2015, Australia, Family@play study^47^	Population: Age: 2 to 3 y Intervention: N = 12; age: 2.85 ± 0.63 y; 60% boys Control: N = 10; age: 2.94 ± 0.61 y; 67% boys Setting: Community	Family‐based activities were undertaken by families and used an anticipatory guidance perspective to facilitate group‐based problem solving to possible challenges. Each session included goal setting specific to each family's circumstances and requirements. A trained facilitator delivered six, 1‐h group sessions each week—total of 5 wk Other behaviours: PA	5 wk	ActivPal time in sitting, standing, and stepping (Janssen et al, 2014). Fifteen‐second epoch. Nonwear time was defined as 10 min of consecutive zero counts and removed from daily wear time. Each participant was required to have at least 6 h of data on each of at least 3 wk and one weekend days to be included in the analysis.	No change in sedentary behaviour (sitting) measured objectively. 1 (95% CI, −7.7 to 9.7), Effect size Cohen's *d* 0.11 Bias corrected (Hedges' g 0.06)
Mendoza et al, 2016, United States, F5K study^42^	Population: Age: 3 to 5 ears Intervention: N = 90, 4.5 ± 0.5 y, 54% male, 100% Latino; neighbourhood disorder 12.1 (4.2)Control: N = 70, 4.4 ± 0.6 y, 50% male, 100% Latino; neighbourhood disorder 14.0 (4.2) Setting: Head start centres	This was a culturally adapted intervention incorporated into the curriculum, which had the overall goal to reduce TV viewing and encourage alternative activities. Modelling provided by preschool teachers, aides, and classmates. Opportunity to rehearse the modelled behaviour to facilitate in the production and retention process. Staff gave feedback to children to reinforce success and gave feedback to children. Parents newsletters with optional home activities. F5K was taught over 7 to 8 wk and consisted of seven themes, each composed of five to six lesson plans of 15 to 30 min, organized around the theme. Other behaviours: none	7 to 8 wk	Actigraph GT1M, 15‐sepoch, nonwear defined as 60 consecutive zero accelerometers count, except for 1 to 2 min of counts between 0 and 100, 3 or more hours of valid wear, cut‐points: less than 37.5 counts/15 s (Pate et al, 2006)	No significant change in objectively measured sedentary time. Sedentary time group × time interaction: −9.5 (95% CI, −23.0 to 4.1), *P* = .172
Nystrom et al, 2017, Sweden, Ministop study^35^	Population: Age: 4.5 ± 0.2 y Intervention: N = 155; age: 4.5 ± 0.1 y; 69% female; Control: N = 158; 4.5 ± 0.1 y; 47% female Setting: Population‐based sample	Smartphone application Other behaviours: diet and PA	6 months	ActiGraph wGT3X‐BT, epoch: 1 s, greater than or equal to 600‐min awake time, validation period unclear, cut‐point. The vector magnitude cut‐offs created by Chandler et al (2015)	No significant difference in sedentary behaviour Intervention: +3.6 ± 48; control: −1.6 ± 55, *P* = .179
O'Dwyer et al, 2012, United Kingdom, Move it!Snapit!Logit!Diary study^37^	Population: Age: 3.8 ± 0.5 y Total: 51.9% male; 91.2% white British; proxy SES—education parents: % high school or less: 63.4%; %technical or trade school: 3.3%; %university:33.3% Intervention: N = 43 Control: N = 33 Setting: Children's centre	Active play‐professional play workers. Educational‐lead researcher and research assistant delivered the intervention. Parents and children received five contact sessions over 10 wk. Sessions of 70 min. Educational workshop, play together. Logbook: self‐monitoring of PA, set graded tasks, provide feedback on performance, contingency rewards, agree behavioural contract. Parents received instructional and educational materials. Text messages support Other behaviour: PA	10 wk	Actigraph GT1M, 5‐s epoch. Nonwear: 20‐min consecutive zeros. 80% of total length of 70% of the sample wore accelerometer (Cattelier et al, 2005). Valid days: 3 d including weekend. Sirard et al (2005) cutpoints	Statistically significant improvement in objectively measured sedentary time Weekday: −8.76 min (95% CI, −12.32 to −5.2); weekend: −23.11 (95% CI, −29.17 to −17.06)
O'Dwyer et al, 2013, United Kingdom^36^	Population: Age: 4.5 ± 0.6 y Total: N = 156, 51.7% male Intervention: N = 70, 4.7 ± 0.5 y; 84.3%white British Control: N = 86, 4.5 ± 0.6 y; 75.3% white British Setting: Preschool	Develop an active curriculum. Manipulated mediators and moderatos of child social environment/target the child's teacher and school environment. Provide staff development and ongoing support to teachers. Six weeks of educational programme to staff and children using. Sessions occurred once per week and lasted 60 min. Staff continue to deliver when professionals left. Comprehensive pack was provided Other behaviour: PA	6 w (follow‐up 6 mo)	ActigraphGT1M, 5‐s epoch. Wear time 80% of total length of 70% of sample, minimum 3 d including one weekend, nonwear 20‐min zero. Sirard et al (2005) cut‐points	No intervention effect for objectively measured sedentary time β = 7.9 (95% CI, −1.5 to 17.3), NS
Østbye et al, 2012, United States, Kids and Adults Now—Defeat Obesity (KAN‐DO) study^43^	Population: Age: 3.06 ± 1.0 y Intervention: N = 200Male: 56% (n = 113) Female: 43.5% (n = 87). Ethnicity: white: 74.5% (N = 149), black: 21.5% (N = 43), other races: 4.0% (N = 8) Household income: Up to $15 000: 9.7 % (N = 19); $15 000 to $30 000 (N = 7.7% (N = 15); $30 001 to $45 000 (N = 9.7% (N = 19); $45 0001 to $60 000 (N = 15.9 (N = 31); $60 001 or more (N = 56.9% (N = 111)Control: N = 200 Male: 55% (n = 110) Female: 45.0% (n = 90) Ethnicity: white: 76% (N = 152), black: 22% (N = 44), other races: 2.0% (N = 4) Household income: up to $15 000: 10.6 % (N = 21); $15 000 to $30 000 (N = 10.1% (N = 20); $30 001 to $45 000 (N = 8.5% (N = 17); $45 0001 to $60 000 (N = 14.6 (N = 29); $60 001 or more (N = 56.3% (N = 112) Setting: Home	Participants received eight interactive family kits that were mailed monthly followed 20‐ to 30‐min supportive telephone counselling based on motivational interviewing techniques. Women were also asked to attend one group session during the 8‐mo period Other behaviours: PA and diet	8 mo	Actical, 6 h/d, 3 valid days, nonwear time 20 min of zeros, less than 12 counts/15‐s epoch (Eveson et al, 2008)	No significant differences in sedentary behaviour. Benjamini‐Hochberg alpha level: 0.042, *P* = .50 NS
Reilly et al, 2006, United Kingdom, MAGIC study^38^	Population: Age: 4.2 ± 0.3 y Intervention: N = 268 (boys = 128, girls = 140); Control: N = 277 (boys = 145, girls = 132) Setting: Childcare	Physical activity at nursery three 30‐min sessions over 24 wk. There was also 6‐wk poster display. The intervention consisted of a nursery element with three 30‐min sessions over 24 wk. And a home element with a resource pack and a health educational leaflet. There were also posters at nursery with focus on physical activity increase through walking and play but also to encourage families to reduce the time spent watching television. At nursery, two members of staff delivered the intervention; at home, it was parents Other behaviour: PA	6 mo (12‐mofollow‐up)	CSA monitor, sedentary no trunk movement, less than 1100/min)	No significant changes in sedentary behaviour. Percentage time spent sedentary 0.08
Tucker et al, 2017, Canada, SPACE study^44^	Population: Age: 2.5 to 4 y Intervention: N = 200; Age: 40.61 ± 7.31 mo; 102 male/98 female; 142% Caucasian, 4% African Canadian, 4% Arboriginal, 3% Arab, 6% Latin‐American, 7% Asian, 19% Other; less than $20 000: 9; $20 000 to $39 999: 14; $40 000 to $59 000: 14; $60 000 to $79 999: 11; $80 000 to $99 999: 16; $100 000 to $119 999: 15; $120 000 to $149 999: 20; greater than $150 000: 45. Control: N = 138; age: 38.72 ± 7.24 mo; 76 male/62 female; 87% Caucasian, 1% African Canadian, 5% Arboriginal, 2% Arab, 3% Latin‐American, 7% Asian, 19% Other; less than $20 000: 11; $20 000 to $39 999: 13; $40 000 to $59 000: 10; $60 000 to $79 999: 12; $80 000 to $99 999: 8; $100 000 to $119 999: 10; $120 000 to $149 999: 11; greater than $150 000: 28 Setting: Childcare	One 4‐h training session; emphasizing the importance of reduction in sedentary behaviour; how to overcome obstacles and to follow the recommendations of the Canadian Sedentary Behaviour guidelines. Other behaviour: PA	8 wk (follow‐up 6 and 12 mo)	Actical, 15‐s epoch, 2 valid days (5‐h wear time), nonwear defined as 20 min of consecutive zeros, greater than or equal to 25 counts (15/s) (Wong et al, 2011)	Sedentary time was significantly lower among preschoolers in the experimental group when comparing postintervention to preintervention, *t*(322) = 2.63, *P* = .009, with no significant effects at follow‐up. Mean difference (postintervention and baseline):−2.13 (95% CI, −3.72 to −0.54)
Verbestel et al, 2015, eight European countries, IDEFICS study^39^	Population: Age: 2 to 9.9 y Boys: N = 3750, age: 6.22 ± 1.88 Girls: N = 3663, age: 6.26 ± 1.79, ethnicity eight European countries Setting: Schools/community	Long‐term community campaign, community environmental and policy interventions, and education of children and parents Other behaviours: Water consumption, increase of fruits and vegetables; increase daily PA, strengthening parent‐child relationship, and adequate sleep	2 y	Actigraph GT1M, 60‐s epoch, 20 consecutive zeros nonwear, 6‐h data, 3 valid days. 20 consecutive zeros nonwear, 6‐h data, 3 valid days, cut‐point: Sedentary less than or equal to 100, 60s^‐1^, Evenson et al (2008)	No change in objectively measured sedentary behaviour. Boys time Xgroup: 0.70, *P* = .175; girls: 0.86, *P* = .096

Abbreviations: CSA, Computer Science and Applications; F5K, Fit5Kids; NS, non significant; PA, physical activity; PAR, physical activity ratio; SED,sedentary behaviour.

### 3.2 Quality assessment

One longitudinal study scored high in the quality assessment,^45^ while the remaining three were of intermediate quality.^34^, ^41^, ^46^ Eight of the intervention studies were considered high quality,^32^, ^33^, ^35^, ^37^, ^38^, ^40^, ^44^, ^47^ including those demonstrating a significant change in sedentary behaviour,^37^, ^44^ and four intervention studies were of intermediate quality.^36^, ^39^, ^42^, ^43^ A description of the quality assessment score of each study is provided in Data S2.

### 3.3 Determinants of sedentary behaviour

Table 4 shows a summary of all identified determinants and the direction and strength of the association combined with the harvest plot.

**Table 4:** Summary of all identified determinants and the direction and strength of the association with harvest plot. Determinants of accelerometer‐assessed sedentary behaviour in young children (less than or equal to 6 y)

Determinants	Decrease Sedentary Time (−)	No Change	Increase Sedentary Time (+)	Studies Showing Association n/N for Row (%)^1^	Summary^2^	Studies Showing Association BCT n/N (%)^3^	Summary Cluster BCT^4^	
Individual (child)								
Longitudinal studies								
Age		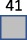		0/1 (0%)	0	NA	NA
Gender		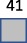		0/1 (0%)	0	NA	NA
Quality of life		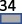		0/1 (0%)	0	NA	NA
Intervention studies								
Shaping knowledge		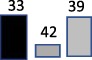		0/3 (0%)	0	0/3 (0%)	0	
Instruction on how to perform behaviour								
Interpersonal (parent/care giver)								
Intervention studies								
Antecedents								
Restructuring the social environment (parents/carers‐child interaction)		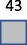		0/1 (0%)	0	0/1 (0%)	0	
Association								
Prompts/cues (parents/carers ‐child interaction)		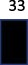		0/1 (0%)	0	0/1 (0%)	0	
Comparison behaviour								
Modelling the behaviour(parents/carers)		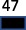		0/1 (0%)	0	0/1 (0%)	0	
Feedback and monitoring								
Other(s) monitoring with awareness (parent/carer‐child interaction)	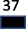	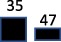		1/3 (33%)	0	1/3 (33%)	0	
Goals and planning								
Behavioural contract (parents/carers‐child interaction)	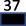			1/1(100%)				
Problem solving/coping planning‐ (parents/carers ‐child interaction)		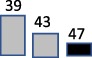		0/3 (0%)	0	1/4 (25%)	00	
Goal setting (behaviour)‐ (parents/carers ‐child interaction)	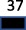	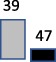		1/3 (33%)	0			
Identity								
Identification of self as a role model (parents/carers)		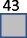		0/1 (0%)	0	0/1 (0%)	0	
Regulation								
Regulate negative emotions (parents/carers)		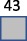		0/1 (0%)	0	0/1 (0%)	0	
Repetition and substitution								
Habit formation (parents/carers)		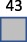		0/1 (0%)	0			
Behaviour substitution (parents/carers‐child interaction)		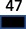		0/1 (0%)				
Graded tasks (parent/carer‐child interaction)	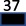			1/1 (100%)	‐	1/3 (33%)	0	
Reward and threat								
Non‐specific reward (parents/carer‐child interaction)	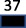	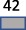		1/2 (50%)	?			
Material reward (parents/carer‐child interaction)	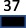	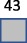		1/2 (50%)	?	1/3 (33%)	0	
Incentive (parents/carer‐child interaction)	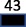			1/1 (100%)	‐			
Self‐belief								
Verbal persuasion to boost self‐efficacy (parents/carers)		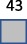		0/1 (0%)	0	0/1 (0%)	0	
Shaping knowledge								
Instruction on how to perform behaviour (parents/carers)		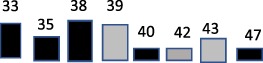		1/9 (11%)	0	1/9 (11%)	00	
Social Support								
Social support (general) (parents/carers)		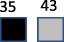		0/2 (0%)	0	0/2 (0%)	0	
Environment (home/preschool/ childcare/community)								
Longitudinal studies								
Time (school time vs out of school time)			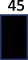	1/1 (100%)	+	NA	NA	
Transition childcare to school			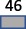	1/1 (100%)	+	NA	NA	
Intervention studies								
Association								
Prompts/cues (preschool teacher‐child interaction)		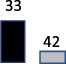		0/2(0%)	0	0/2	0	
Antecedents								
Restructuring physical environment (preschool environment)	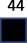	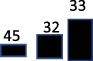		1/4 (25%)	00			
Restructuring physical environment (home)		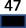		0/1 (0%)	0			
Restructuring physical environment (community)		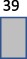		0/1 (0%)	0	1/6 (17%)	00	
Restructuring social environment (preschool environment)	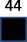	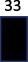		1/2 (50%)	?			
Repetition and substitution								
Behaviour substitution (preschool teacher child interaction)		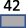		0/1 (100%)	0			
Habit formation (preschool teacher child interaction)		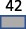		0/1 (100%)	0	0/1 (0%)	0	
Comparison behaviour								
Modelling the behaviour (preschool teachers)		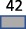		0/1 (0%)	0	0/1 (0%)	0	
Feedback and monitoring								
Other(s) monitoring with awareness (preschool teacher‐child interaction)		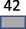		0/1 (0%)	0	0/1 (0%)	0	
Reward and threat								
Non‐specific reward (preschool curriculum)		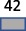		0/1 (0%)	0	0/1 (0%)	0	
Shaping knowledge								
Instruction on how to perform behaviour (childcare/preschool teachers)	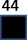	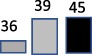		1/4 (25%)	00			
Instruction on how to perform behaviour (community)		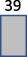		0/1 (0%)	0	1/4 (20%)	00	
Social Support								
Social support (general) (preschool teacher)		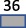		0/1 (0%)	0	0/1 (0%)	0	

Abbreviation: BCT, behaviour change technique; NA, not applicable.

1 n = number of studies which support the direction of association; N= total number of studies which investigated the association

2 Overall summary of findings for each outcome with the direction of association.

3 Number of studies which support the association of the grouping behaviour change technique

4 Overall summary of findings for the grouping behaviour change technique

Notes: Bar charts were displayed as follow: 1. Position based on direction of association (decrease in sedentary behaviour (−), no change in sedentary behaviour, increase in sedentary behaviour (+); 2. Height of bar represented size of study (short <300 participants, medium 300–500 participants, high >300 participants); 3. Colour of bar representing quality: black, dark grey and white with darker bars representing higher quality studies; 4. Symbol on top show study for identification.

#### 3.3.1 Longitudinal studies

Five determinants of sedentary behaviour were identified in longitudinal studies. At the individual level determinants such as age, gender, and quality of life were not associated with sedentary behaviour. However, at the environmental level, the after childcare/school period^45^ (sedentary time outside childcare/school period) and children's transition from childcare (as a period before starting full‐time formal schooling) to formal schooling^46^ were positively associated with (an increase in) sedentary behaviour in young children.

#### 3.3.2 Intervention studies

Only one intervention study targeted all the levels of the socioecological model^39^ (Table 5). Two of the intervention studies^33^, ^42^ 1targeted three levels (ie, individual, intrapersonal, and environmental levels), while two^40^, ^47^ targeted two levels (ie, intrapersonal and environmental levels). Seven studies only targeted one level of the socioecological model, namely, interpersonal^35^, ^37^, ^38^, ^43^ or environment.^32^, ^36^, ^44^

**Table 5:** Determinants of sedentary behaviour, BCT and level targeted (socio‐ecological model) for intervention studies.

Author (year)	Determinant	Cluster of BCT Component of BCT Target Population	Level targeted According to Socioecological Model (Group Targeted)
Adamo et al (2017)^40^	Starter kit equipment	1. Antecedents a. Restructuring the physical environment i. Childcare environment	Interpersonal (parents/care giver) Environment (childcare)
	Workshops training sessions and biweekly booster session to childcare providers Training material provided to parents, webinar, postcards	2. Shaping knowledge a. Instruction on how to perform a behaviour i. Parents/care giver ii. Childcare staff	
Cardon et al (2009)^32^	Intervention: play equipment provided at break time, marking painted on playground	1. Antecedents a. Restructuring the physical environment i. Preschool environment	Environment (preschool)
De Craemer et al (2016)^33^	Preschool environment change (eg, standing play stations, use the hallway, and movement corners) Longer movement breaks Doing activities while standing	1. Antecedents a. Restructuring physical environment i. Preschool environment b. Restructuring social environment i. Preschool environment	Individual (child) Interpersonal (parents/care giver) Environment (preschool)
	Poster including key messages to decrease sedentary behaviour given to parents (eg, don't sit down for a long time, get up and be active, do not eat in front of the screen, limit screen viewing activities, and include active movement breaks in the children's daily lives) No TV‐signs‐Weekly calendar in preschool	2. Association a. Prompt/cues i. Parents/care giver ii. Preschool environment	
	Stories to children (kangaroo stories and kangaroo as a mascot Parents newsletter (eg, general information about sedentary behaviour; guidelines regarding screen time and sedentary behaviour; tips to limit children's time spent sedentary and screen time, tips for movement breaks, and parents are a role model) Tip‐cards (eg, how to motivate the child; how to decrease screen‐related activities; and parent‐child activities)	3. Shaping knowledge a. Instructions on how to perform behaviour i. Children ii. Parents/care giver	
Hinkley et al (2015)^47^	Strategies—safe place in home, no TV in bedroom, fewer TVs home Strategies—decrease parent electronic media	1. Antecedents a. Restructuring physical environment i. Home 2. Comparison of behaviour a. Modelling of the behaviour i. Parents/care giver	Interpersonal (parents/care giver) Environment (home)
	Monitoring and remonitoring when necessary	3. Feedback and monitoring‐ a. Other(s) monitoring with awareness i. Parents/care giver	
	Strategies—setting rules, planning (for normal and unusual days), challenge identification and problem solving Goal setting (record goals and review). Super parents/carers challenge—no electronic media for entertainment for the whole parents/carers for the whole week	4. Goals and planning a. Problem solving/copying planning i. Parents/care giver b. Goal setting (behaviour) i. Parents/care giver	
	Strategies given to parents to help children be active instead	5. Repetition and substitution a. Behaviour substitution i. Parents/care giver	
	Raise awareness and recognize benefits	6. Shaping knowledge a. Instruction on how to perform a behaviour i. Parents/care giver	
Mendoza et al (2016)^42^	Reinforcement through proximal cues	1. Associations a. Prompts/cues i. Preschool teachers	Individual (child) Interpersonal (parents/care giver) Environment (preschool)
	Modelling provided by preschool teachers, aides, and classmates	2. Comparison behaviour a. Modelling of the behaviour i. Preschool staff	
	Feedback to children	3. Feedback and monitoring a. Other(s) monitoring with awareness i. Preschool teacher	
	Encourage alternative activities Rehearse the modelled behaviour	4. Repetition and substitution a. Behaviour substitution i. Preschool teacher b. Habit formation children i. Preschool teacher	
	Rewards incorporated into the curriculum	5. Reward and threat a. Non‐specific reward i. Preschool curriculum	
	Educational curriculum Parents newsletters	6. Shaping knowledge a. Instructions on how to perform behaviour i. Children ii. Parents/care giver	
Nystrom et al (2017)^35^	Parents were asked to provide information about sedentary behaviour once a week and provided with a graphic feedback	1. Feedback and monitoring a. Other(s) monitoring with awareness i. Parents/care giver	Interpersonal (parents/care giver)
	Parents could contact a psychologist to ask questions	2. Social support a. Social support (general) i. Parents/care giver	
	Smartphone intervention included—12 themes were introduced biweekly including sedentary time. Intervention contained general information, advice, and strategies to change behaviour to parents	3. Shaping knowledge a. Instruction on how to perform a behaviour i. Parents/caregiver.	
O'Dwyer et al (2012)^37^	Parents log book for self‐monitoring	1. Feedback and monitoring a. Other(s) monitoring with awareness i. Parents/care giver	Interpersonal (parents/care giver)
	Parents log book for agree to a behavioural contract Parents log book for goal setting and review of behavioural goals	2. Goals and planning a. Behavioural contract i. Parents/care giver b. Goal setting (behaviour) i. Parents/care giver	
	Completed log books were linked to a progressive reward system linked to physical activity promotion Parents log book for contingent rewards After completion of all posttest data collection, families received a certificate, active play key fob and a activity song book Parents log book to set graded tasks	3. Reward and threat a. Incentive i. Parents/care giver b. Material reward i. Parents/care giver c. Non‐specific reward i. Parents/care giver	
	Parents workshop—guidelines, discuss alternatives, and instructional materials.	4. Repetition and substitution a. Graded tasks i. Parents/caregiver	
	Parents log book for provide instruction for behaviour tasks and contained contact details for additional support. Families received text messages between each intervention session to communicate key messages	5. Shaping knowledge a. Instructions on how to perform a behaviour i. Parents/care giver	
O'Dwyer et al (2013)^36^	Train staff to deliver active curriculum, full active play programme Staff development	1. Shaping knowledge a. Instructions on how to perform a behaviour i. Preschool teachers	Environment (preschool)
	Ongoing support to preschool teachers	2. Social support a. Social support (general) i. Preschool teachers	
Østbye et al (2012)^43^	A supportive home environment	1. Antecedents a. Restructuring the social environment i. Parents/care giver	Interpersonal (parents/care giver)
	Barriers to change behaviour	2.Goal and planning a. Problem solving—coping planning i. Parents/care giver	
	Parents as role modelling	3. Identity a. Identification of self as a role model i. Parents/care giver	
	Target parent emotion regulation Stress management	4. Regulation a. Regulate negative emotions i. Parents/care giver	
	Rewards to reinforce behaviour including: chart, yoga mat, pedometer, portion plate	5. Reward and threat a. Material reward i. Parents/care giver	
	Reinforced content from the parents/carers kits and set aside time for role play and group discussion.	6. Repetition and substitution a. Habit formation i. Parents/care giver	
	Motivation self‐efficacy	7. Self‐belief a. Verbal persuasion to boost self‐ efficacy i. Parents/care giver	
	Motivational interviewing mother	8. Social support a. Social support (general) i. Parents/care giver	
	Education health behaviours. Parenting skills instruction—authoritative parenting style	9. Shaping knowledge a. Instruction on how to perform a behaviour, i. Parents/care giver	
Reilly et al (2006)^38^	Resource pack to encourage families to seek opportunities to reduce the time spent watching television	1. Shaping knowledge a. Instruction on how to perform a behaviour i. Parents/care giver	Interpersonal (parents/care giver)
Tucker et al (2017)^44^	Environment modifications (eg, portable equipment) Restructuring outdoor playtime (two 60 min into four 30 min)	1. Antecedents a. Restructuring the physical environment i. Childcare environment b. Restructuring the social environment i. Childcare environment	Environment (childcare)
	Staff and directors training about importance of reducing sedentary time, recommendations for overcoming obstacles, provided examples of activities that could be implemented in childcare	2. Shaping knowledge a. Instruction on how to perform a behaviour i. Childcare providers.	
Verbestel et al (2015)^39^	Community environmental and policy interventions (eg, play streets and community playgrounds).	1. Antecedents a. Restructuring the physical environment i. Community	Individual (child) Interpersonal (parents/care giver) Environment (schools, community) Policy (community infrastructure)
	Parents/carers materials also contained strategies to remove barriers and facilitate their ability to create health promoting. Each healthy week, a specific behavioural objective was handled.	2. Goals and planning a. Problem solving/coping planning i. Parents/care giver b. Goal setting (behaviour) i. Parents/care giver	
	Long‐term community media campaign, education of children and parents. Parents/carers target module consisting of educational materials (posters and flyers) School community group: implement modules at school level. Educational materials were distributed through the school and the community.	3. Shaping knowledge a. Instruction on how to perform a behaviour i. Children ii. Parents/care giver iii. Schools, community	

Interventions targeted an average of 3.6 (*SD* 2.4) BCT clusters. At the level of BCT components, 21 were targeted. The most commonly included BCT cluster was “shaping knowledge”: within this “instruction on how to perform a behaviour” was the most frequently targeted BCT component (11 out of 12 studies).^33^, ^35^, ^36^, ^37^, ^38^, ^39^, ^40^, ^42^, ^43^, ^44^, ^47^ “Shaping knowledge” was targeted at all levels of the social ecological model, although the majority of studies targeted it at the intrapersonal level (nine out of 12 studies).^33^, ^35^, ^37^, ^38^, ^39^, ^40^, ^42^, ^43^, ^47^ The BCT cluster “Antecedents” was included in eight out of 12 studies,^32^, ^33^, ^39^, ^40^, ^42^, ^43^, ^44^, ^47^ particularly the BCT component “restructuring physical environment” at preschools (five out of 12 studies).^32^, ^33^, ^40^, ^44^, ^47^

Only three BCT components were identified as determinants of decreases in sedentary behaviour. These included “behavioural contract” (cluster—“goals and planning”), “graded tasks” (cluster—“repetition and substitution”), and “incentive” (cluster—“reward and treat”). However, these determinants were extracted from a single high‐quality study with a small sample size (N = 43 intervention and N = 33 control).^37^ There was inconsistent evidence for “non‐specific reward and material reward” (cluster—“reward and treat”) as determinants of sedentary behaviour.

## 4 DISCUSSION

### 4.1 Main findings

This systematic review is the first to synthesize the evidence on determinants of change in accelerometer‐assessed sedentary behaviour in preschool‐aged children. Five determinants were investigated in four longitudinal studies and 21 determinants (ie, BCT components) in 12 intervention studies. These determinants spanned all levels of the socioecological model. Only “instruction on how to perform a behaviour” at both the interpersonal and environmental level, and “restructuring physical environment,” were identified in four or more studies, but neither were associated with behaviour change.

Evidence from longitudinal studies showed that the outside childcare/school period^45^ and transition from childcare (ie, a period when children have not yet started formal school) to formal schooling^46^ were associated with increases in sedentary behaviour in young children. This suggests that targeting relevant policies and practices with respect to sedentary behaviours at schools may be important.

The findings gathered from intervention studies suggest that “behavioural contracts” (BCT cluster—“goals and planning”), “graded tasks” (BCT cluster—“repetition and substitution”), and “incentives” (BCT cluster—“reward and treat”) were associated with decreases in sedentary behaviour. However, these determinants were only identified in one study each. According to the Behaviour Change Taxonomy (v1),^26^ “behavioural contracts” are when a targeted behaviour is specified, written and signed in a contract, agreed by one person, and witnessed by another. For “graded tasks” individuals are initially set easy to perform tasks and are then challenged to progress at achievable levels until the behaviour is performed. Finally, for “incentives,” participants are informed that a reward will be delivered only if there has been an effort (or progress) in achieving a behaviour.

It is important to note that although only two intervention studies^37^, ^44^ showed statistically significant reductions in accelerometer‐assessed total sedentary behaviour, four others studies included here found a significant decrease in screen‐viewing behaviour.^33^, ^42^, ^43^, ^47^ In two studies,^42^, ^47^ there was a reduction in electronic media use^47^ or TV viewing,^42^ while in the others,^33^, ^43^ although there was no effect on total TV viewing, there was a subgroup effect (ie, girls reduction in TV viewing on weekends)^33^ and changes in parenting outcomes related to TV viewing (ie, TV snacks and dinner in front of TV).^43^

### 4.2 Findings in context of previous research

In this review, we found evidence, although limited,^45^ that the outside childcare/school period might be a potential determinant of sedentary behaviour in young children. Similar findings were observed in older children, with the after‐school period shown to be associated with an increase in accelerometer‐assessed sedentary time and TV viewing.^57^, ^58^ It has been argued that the after‐school period has a large impact on children's accumulation of sedentary behaviour, and a small change in after‐school sedentary behaviour might have a large impact on daily sedentary time.^57^ Interestingly, the transition from childcare to formal schooling was shown here to be associated with increases in sedentary time in young children,^46^ with children being more sedentary after starting primary school. This suggests that the formal school environment may foster more sedentary behaviours, as compared with childcare.

A number of determinants at the individual level were not associated with change in sedentary behaviour, including age, which was only assessed in one medium size, intermediate quality study.^41^ It does contradict findings from a previous systematic review that found age as strong determinant of sedentary behaviour in youth (less than 18 years).^16^ However, this may be because of the limited age range of participants included in studies conducted in early years, which restricts the opportunity to investigate this exposure as a determinant.

By focusing on the key ingredients of interventions, the BCTs identified in this review might help to inform future interventions to aid longer term behaviour change in young children. “Behavioural contracts” have been shown previously to positively impact physical activity for older adult populations and disease‐specific conditions.^59^, ^60^ There is, however, limited evidence on younger and healthy populations. One example is an adolescent‐targeted intervention that used behavioural contract in addition to other intervention features, which was successful in reducing screen‐time in the intervention group, although no between‐group differences were observed.^61^ Likewise, the BCT “graded tasks” has predominantly been used in adults. A previous systematic review found that implementing “graded tasks” was associated with successful outcomes in longer term when promoting physical activity and healthy eating in adults with overweight and obesity.^59^ Evidence in young children is however limited. Finally, while “incentives” appear to support change in behaviour in adults,^62^, ^63^, ^64^ few studies have investigated the effect on behaviour change in children and those that have focussed primarily on diet in children at school age.^65^, ^66^ In the studies identified in this review,^37^, ^42^, ^43^ different forms of incentives were delivered under the “reward and threat” cluster including “incentives (outcomes),” “material reward,” “social reward,” and “non‐specific reward.” Only “incentive (outcome)” (definition according to Michie et al^26^ “inform that a reward will be delivered if and only if there has been effort and/or progress in achieving the behavioural outcome”) was successful.^37^

Previous review‐level evidence^21^ highlighted parental monitoring as a determinant of physical activity in young children. The same systematic review found that provider training was moderately associated with vigorous physical activity; however, child and parental knowledge was consistently not associated. In this study, we found that “shaping knowledge” (BCT component—“instruction on how to perform a behaviour”) was not associated with changes in sedentary behaviour at all levels of the socioecological model. This reflects findings (ie, child and parental knowledge) of the previous physical activity review^21^; however, shaping knowledge at childcare and preschool level is not associated with changes in sedentary behaviour.

Elements of the physical environment have been frequently investigated as a determinant of physical activity and sedentary behaviour.^16^, ^21^ Similar to our systematic review, a previous review that focused on determinants of physical activity found that restructuring the physical environment in preschool did not lead to changes of this behaviour in early years.^21^ Likewise, another systematic review on the determinants of sedentary behaviour in youth^16^ found that although environmental determinants were explored in a large number of studies, few found an association with sedentary behaviour.

### 4.3 Strengths and limitations

To our knowledge, this is the first systematic review to assess the determinants of change in sedentary behaviour in young children. The use of accelerometer‐assessed sedentary time is a key strength, as self‐report measures tend to focus on TV or screen viewing, which has been shown to have low validity to measure total sedentary time.^18^, ^20^ However, it can also be seen as a limitation as self/proxy report measures provide contextual information (ie, setting and type of activity) about sedentary behaviour that provide valuable information about sedentary activities undertaken by young children.^67^

No time or language restrictions were applied, ensuring high sensitivity in identifying the literature. However, it is possible that all relevant publications were not included, and publication bias cannot be ruled out. Moreover, although all effort was made to extract information of intervention features (and therefore determinants) from relevant documents (ie, protocols, trial registers, supplementary files, and additional papers), it was not always possible to detail the exact intervention elements for all studies.^68^ Furthermore, it is possible that the intervention strategies embedded in the included studies were not captured by the coding of BCT taxonomy if these were not clear or sufficiently precise. The use of template for intervention description and replication (TIDieR) checklist to specify essential elements of the intervention and the use of the BCT taxonomy coding in future studies might help better identifying elements of interventions in future^68^ and facilitate evidence synthesis that could guide implementation.^69^

Despite substantial heterogeneity in the included studies, exposure and outcome measures, the combined used of summary tables to assess consistency of associations across studies,^30^ and the use of Harvest plot^31^ enabled us to provide a detailed summary of findings. Although we included a limited the number of studies, they were of intermediate (n = 7) and high (n = 9) quality, strengthening the findings reported here. As the majority were intervention studies, this highlights a lack of high‐quality longitudinal observational research in this age group. Moreover, all studies were conducted in high‐income countries, and findings cannot therefore be generalized to low‐ and middle‐income countries.

### 4.4 Recommendations for policy and practice

Although several interventions have been developed to target sedentary behaviour in childcare/school setting,^70^, ^71^ it appears that the after school is a period of high prevalence in sedentary behaviour.^72^ This suggests that more needs be done to prevent sedentary behaviour in the home environment.^73^ Similarly, childcare settings may be more supportive than the formal school setting for reducing sedentary behaviour. The more structured curriculum in primary schools may reinforce sedentary behaviour; therefore, initiatives to reduce sitting time such as classroom‐based physical activity^74^ or standing desks^75^ might be good strategies to be implemented at schools.

Also, from this review, we found strong evidence that shaping knowledge (instruction on how to perform a behaviour) at individual, parents/carers, and at childcare/preschool environment is not sufficient to change sedentary behaviour of young children. Therefore, we recommend that in practice, instruction on how to perform a behaviour should not be delivered in isolation, as it might not bring the expected benefits on the reduction of sedentary behaviour.

Similar findings we observed on restructuring the environment, as it seems that when this BCT component is implemented by its own^32^ or in some cases in combination with other BCTs,^33^, ^40^, ^43^, ^47^ it does not promote the expected reductions in sedentary behaviour. However, more evidence is needed.

Interpersonal determinants such as having “behavioural contract” (cluster “goals and planning”), promoting “graded tasks” (cluster “repetition and substitution”), and receiving “incentives” (cluster “reward and treat”) might be appropriate behavioural strategies to be incorporated into sedentary behaviour interventions in young children. Although more evidence is needed, interventions may benefit from incorporating other BCT components in the cluster of “goals and planning,” “repetition and substitution,” and “reward and treat.”

## 5 CONCLUSION

We identified limited evidence on the determinants of change in accelerometer‐assessed sedentary time in children 0 to 6 years. The available evidence suggests that the after childcare/school period and transition from childcare to formal school are potential determinants. Furthermore, the following determinants at the interpersonal level were associated with a decrease in sedentary behaviour: goals and planning (ie, behavioural contract), repetition and substitution (ie, graded tasks), and reward and treat (ie, incentives). More longitudinal and intervention research is needed to provide more robust evidence on the determinants of sedentary behaviour in children, to in turn inform the development of effective interventions.

## FUNDING INFORMATION

This work was additionally supported by the Medical Research Council (Unit Programme number MC_UU_12015/7) and the Centre for Diet and Activity Research (CEDAR), a UKCRC Public Health Research Centre of Excellence. The British Heart Foundation, Cancer Research UK, Economic and Social Research Council, Medical Research Council, the National Institute for Health Research, and the Wellcome Trust, under the auspices of the UK Clinical Research Collaboration, provided funding (CEDAR grant numbers: ES/G007462/1; 087636/Z/08/Z; MR/K023187/1). KRH is funded by the Wellcome Trust (107337/Z/15/Z).

## CONFLICT OF INTEREST

No conflict of interest was declared.

## Supporting information

Data S1: An example of search strategy ‐ MEDLINE and Embase.

Data S2: Summary of the included longitudinal studies.
