# Supplementary material for: Determinants of change in accelerometer‐assessed sedentary behaviour in children 0 to 6 years of age: A systematic review
Source: Obes Rev. 2019 Jun 27;20(10):1441–64. doi: 10.1111/obr.12882 (PMC6772060; doi:10.1111/obr.12882)
Supplement: Supplementary file 1 — Data S1: An example of search strategy ‐ MEDLINE and Embase. [file OBR-20-1441-s001.docx]

Supplement 1: an example of search strategy - MEDLINE and Embase

Last run: 23/11/2016

Update searches run: 27-03-2018

Medline and Embase (Embase 1996 to 2018 Week 13; Ovid MEDLINE(R) 2014 to March Week 3 2018)

| 1 | (Determin*4 or correlates or factors or predict*3 or associat*3 or interaction or influence*1 or prevent*3 or reduc*5 or increas*3 or promot*3 or education or curriculum or program*3 or polic*3 or media or campaign or review or intervention*1 or initiative*1 or strategy*3 or evaluation or trial).mp. | 17769087 |
| --- | --- | --- |
| 2 | (Infant* or Toddler* or Preschool* or Nurser*).mp. | 886614 |
| 3 | (inactiv*3 or TV or Television or Tele or sedentary or (screen adj time) or ((computer or laptop) adj time) or (sitting adj time) or video$gam* or (media adj ("use" or exposure)) or (mobile adj (phone or device)) or (electronic adj (tablet or notebook))).mp. | 201287 |
| 4 | 1 and 2 and 3 | 5449 |
| 5 | 4 not (cerebral palsy or asthma or cystic fibrosis or autism).mp. | 5274 |

*Medline: 1117*

*Embase: 4157*
