# Supplementary material for: Determinants of change in accelerometer‐assessed sedentary behaviour in children 0 to 6 years of age: A systematic review
Source: Obes Rev. 2019 Jun 27;20(10):1441–64. doi: 10.1111/obr.12882 (PMC6772060; doi:10.1111/obr.12882)
Supplement: Supplementary file 2 — Data S2: Summary of the included longitudinal studies. [file OBR-20-1441-s002.docx]

Supplementary file 2: Quality assessment score for the included longitudinal and intervention studies.

Table 1: Quality assessment longitudinal studies

| **Author, year** | **More than 50 participants analysed** | **Studies representing general population** | **Prospective study design** | **Adjusted/multivariate analysis** | **Objective measure of outcome** | **Objective measure of exposure** | **Quality criteria met (score)** |
| --- | --- | --- | --- | --- | --- | --- | --- |
| Arundel et al. 2013 [45] | Low risk | Low risk | Low risk | High risk | Low risk | Low risk | 5/6  (high) |
| Carson et al. 2016[46] | Low risk | High risk | Low risk | High risk | Low risk | Low risk | 4/6 (intermediate) |
| Janz et al. 2005[41] | Low risk | High risk | Low risk | High risk | Low risk | Low risk | 4/6  (intermediate) |
| Michels et al., 2016 [34] | Low risk | High risk | Low risk | Low risk | Low risk | High risk | 4/6 (intermediate) |

Table 2: Quality assessment intervention studies

| **Author, year** | **Randomisation** | **Effect of intervention reported for all outcomes** | **Pre-intervention data on all outcomes** | **Post intervention data on all outcomes** | **Allocation concealment** | **Blinding** | **Objective measurement of outcome** | **Retention>70%** | **Quality criteria met (score)** |
| --- | --- | --- | --- | --- | --- | --- | --- | --- | --- |
| Adamo et al. 2017 [40] | low risk | low risk | low risk | low risk | low risk | High risk | low risk | high risk | 6/8 (high) |
| Cardon et al. 2009 [32] | Low risk | Low risk | Low risk | Low risk | High risk | High risk | Low risk | Low risk | 6/8 (high) |
| De Craemer et al. 2016 [33] | Low risk | Low risk | Low risk | Low risk | Low risk | High risk | Low risk | Low risk | 7/8 (high) |
| Hinkley et al. 2015 [47] | Low risk | Low risk | Low risk | Low risk | Low risk | Low risk | Low risk | Low risk | 8/8 (high) |
| Mendoza et al. 2016 [42] | Low risk | High risk | High risk | High risk | High risk | High risk | Low risk | Low risk | 3/8 (intermediate) |
| Nystrom et al. 2017 [35] | Low risk | Low risk | Low risk | High risk | Low risk | High risk | Low risk | Low risk | 6/8 (high) |
| O’Dwyer et al., 2012 [37] | Low risk | Low risk | Low risk | Low risk | High risk | High risk | Low risk | Low risk | 6/8 (high) |
| O’Dwyer et al. 2013 [36] | Low risk | Low risk | High risk | High risk | High risk | High risk | Low risk | Low risk | 4/8 (intermediate) |
| Østbye et al. 2012 [43] | Low risk | Low risk | Low risk | Low risk | High risk | High risk | Low risk | High risk | 5/8 (intermediate) |
| Reilly et al., 2006 [38] | Low risk | Low risk | Low risk | Low risk | High risk | High risk | Low risk | Low risk | 6/8 (high) |
| Tucker et al. 2017 [44] | Low risk | Low risk | Low risk | Low risk | Low risk | High risk | Low risk | Low risk | 7/8 (high) |
| Verbestel et al. 2015 [39] | Low risk | Low risk | Low risk | High risk | High risk | High risk | Low risk | Unsure | 4/8 (Intermediate) |
